# Transitions in Immunoassay Leading to Next-Generation Lateral Flow Assays and Future Prospects

**DOI:** 10.3390/biomedicines12102268

**Published:** 2024-10-06

**Authors:** Koyu Fujiuchi, Noriko Aoki, Tetsurou Ohtake, Toshihide Iwashita, Hideya Kawasaki

**Affiliations:** 1 NanoSuit Research Laboratory, Institute of Photonics Medicine, Division of Preeminent Bioimaging Research, Hamamatsu University School of Medicine, Hamamatsu 431-3125, Japan; e23001@hama-med.ac.jp; 2 Research and Development Department, TAUNS Laboratories, Inc., Izunokuni-shi 410-2325, Japan; n_aoki@tauns.co.jp (N.A.); t_ohtake@tauns.co.jp (T.O.); 3 Department of Regenerative and Infectious Pathology, Hamamatsu University School of Medicine, Hamamatsu 431-3125, Japan; toshiiwa@hama-med.ac.jp

**Keywords:** lateral flow assay, point-of-care testing, future technology, diagnostic device

## Abstract

In the field of clinical testing, the traditional focus has been on the development of large-scale analysis equipment designed to process high volumes of samples with fully automatic and high-sensitivity measurements. However, there has been a growing demand in recent years for the development of analytical reagents tailored to point-of-care testing (POCT), which does not necessitate a specific location or specialized operator. This trend is epitomized using the lateral flow assay (LFA), which became a cornerstone during the 2019 pandemic due to its simplicity, speed of delivering results—within about 10 min from minimal sample concentrations—and user-friendly design. LFAs, with their paper-based construction, combine cost-effectiveness with ease of disposal, addressing both budgetary and environmental concerns comprehensively. Despite their compact size, LFAs encapsulate a wealth of technological ingenuity, embodying years of research and development. Current research is dedicated to further evolving LFA technology, paving the way for the next generation of diagnostic devices. These advancements aim to redefine accessibility, empower individuals, and enhance responsiveness to public health challenges. The future of LFAs, now unfolding, promises even greater integration into routine health management and emergency responses, underscoring their critical role in the evolution of decentralized and patient-centric healthcare solutions. In this review, the historical development of LFA and several of the latest LFA technologies using catalytic amplification, surface-enhanced Raman scattering, heat detection, electron chemical detections, magnetoresistance, and detection of reflected electrons detection are introduced to inspire readers for future research and development.

## 1. Introduction

In current clinical settings, systems and reagents that allow the rapid acquisition of on-site test data have become the norm [1,2,3]. The use of point-of-care testing (POCT) products enables real-time testing at the patient’s side, contributing significantly to rapid decision-making by physicians [4]. The concept of POCT originated as early as the 1990s in the UK and USA, and with the development of platforms that embody this concept, it has become widely pervasive in clinical settings [5,6,7]. POCT products have undergone rapid technological advancements since their inception. Currently, various POCT products are available for pregnancy tests, blood gases, biochemistry, glucose level, infectious diseases, coagulation tests, and cardiac markers [8,9,10,11].

In particular, the lateral flow assay (LFA), which is based on the principle of immunochromatography, has become a key tool in infection control owing to its simplicity, combined with the outbreak of the pandemic caused by SARS-CoV-2 in 2019 [12,13,14,15]. Currently, it is used as an immunodiagnostic reagent that can be handled by nonmedical professionals. The advent of LFA reagents that allow “anyone, anywhere, affordably, and quickly” to detect targets has been accompanied by the introduction and evolution of various immunodiagnostic methods. This has helped establish the current platform for POCT reagents.

We have observed the evolution of measurement techniques based on antigen–antibody reactions. Tracing back its history, in 1936, M. Heidelberger and others attempted to analyze antigens using precipitin (an antibody that reacts with antigens to form a precipitate). However, accurate concentration measurements were not obtained [16]. In 1959, Yalow and Berson reported a radioimmunoassay (RIA) that combines radioactive isotopes with antibodies [17]. This assay was used to measure insulin levels. This method used a competitive process to measure radiolabeled antigens. While highly sensitive, RIA had drawbacks, such as a limited measurement range, the creation of radioactive waste, and the need for specialized equipment and training. Due to these demanding requirements, reagents that are easier to handle in general clinical testing settings are preferred.

To overcome these disadvantages, technology based on enzyme-linked immunosorbent assays (ELISA) using enzyme labels instead of radioactive labeling have gained prominence [18,19,20,21]. Initially, measurements were made using colorimetric quantification based on absorbance; however, with the development of luminescent substrates, higher sensitivity was achieved [21]. However, ELISA are not widely used in general medical practice because of the time required to obtain results (2–3 h), reagent stability (requiring refrigeration), transportation costs, and ease of use.

The fundamental principle of immobilizing antibodies and detecting antigens using the sandwich method remains the same; however, the technology for detecting immune complexes on membranes was established in the 1970s and 1980s. Immunochromatographic assays based on membranes primarily composed of nitrocellulose have been commercialized as immunodiagnostic reagents by several manufacturers, including Becton Dickinson [22]. This technology was introduced to the market and became widely used in rapid diagnostic tests. Immunochromatographic methods are divided into two main types: vertical flow and lateral flow. In the vertical flow type, the reaction solution containing the antigen moves vertically, while in the lateral flow type, the test substance and reagents move horizontally.

Currently, reagents of the vertical flow immunoassay (VFA) type are almost extinct, and LFA reagents are predominantly used. The LFA has become synonymous with rapid testing in clinical settings, primarily because of its user-friendliness. The VFA involves the same sample collection step; however, it requires a reaction between the analyte and labeled antibodies, followed by the deployment of reagents onto a membrane to conduct a sandwich reaction, and sometimes washing steps as needed. As it involves multiple steps, the VFA takes longer to produce results than the LFA, and the measurement process becomes more complex [23]. Additionally, owing to their structure, VFAs may not allow all the labeled antibodies to contact membrane-bound antibodies. It has been speculated that the LFA is more sensitive than the VFA because the antigen solution flows in one direction into an area with fixed capture antibodies.

Compared to the VFA, which involves multiple steps to achieve an antigen–antibody reaction, the LFA operates elegantly in a single step. Additionally, the results are obtained in just a few minutes to approximately 10 min. The LFA, which is simple, fast, and cost-effective, has been widely used in clinical settings since its launch to the present day.

The global pandemic caused by SARS-CoV-2 in 2019 led to the widespread recognition and use of the LFA, which was already a standard test in clinical settings, among the general public, not just medical professionals. This review aims to inform readers about the technical evolution of the immunochromatography method, as well as the current development status and emerging LFA platforms.

## 2. Basic Principle and Structure of LFA

**Figure 1:**
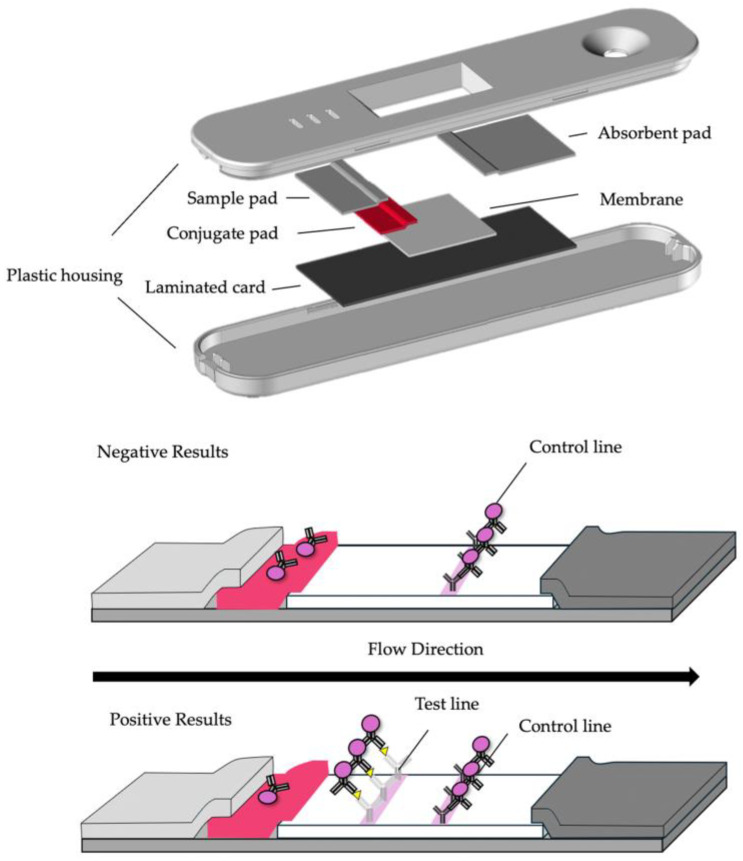
Overview of a typical LFA as a point-of-care diagnostic device. This device typically consists of a nitrocellulose membrane, sample pad, conjugate pad, absorbent pad, and plastic housing (upper image). The lower images provide a schematic representation of positive and negative results of LFA using the sandwich method.

The basic principles of the LFA reagent are illustrated in Figure 1. The reagent generally consists of a nitrocellulose membrane, sample pad, conjugate pad, absorbent pad, and plastic housing. Typically, two types of specific antibodies are used to detect the target. In cases where the sandwich method is employed for detection, one antibody is immobilized on the membrane. The second antibody is immobilized onto a label and impregnated into a conjugate pad. Common labels include metal particles, colored latex particles, fluorescent particles, quantum dots, upconversion particles (which absorb low-energy light, like infrared light, and emit higher-energy light, such as visible light), and enzymes. Essentially, this immunodiagnostic reagent allows the visual detection of the presence or absence of the target.

The immunoassay begins by allowing the test sample extracted from the specimen collected from the subject or patient to flow onto a sample pad. The insoluble particles in the extract are filtered on the sample pad, whereas the antigen solution permeates the conjugate pad. Subsequently, the antigen reacts with the labeled antibodies as it moves towards the nitrocellulose membrane. An antigen–antibody reaction occurs on the nitrocellulose membrane; hence, a membrane with smaller pore sizes is used to enhance sensitivity. This is because the smaller pore size slows down the flow rate, increasing the opportunities for contact between the antibody and antigen. However, the pore size of a nitrocellulose membrane is typically more than 10 times the diameter of the labeled particles.

When the antigen-bound labeled antibodies are captured by the antibodies immobilized on the test line, a sandwich complex is formed that appears as a line. The control line is typically coated with antibodies that capture the labeled particles and is used to indicate that the labeled antibodies have passed over the test line. The reaction fluid flowing over the nitrocellulose membrane ultimately reaches the absorbent pad.

**Figure 2:**
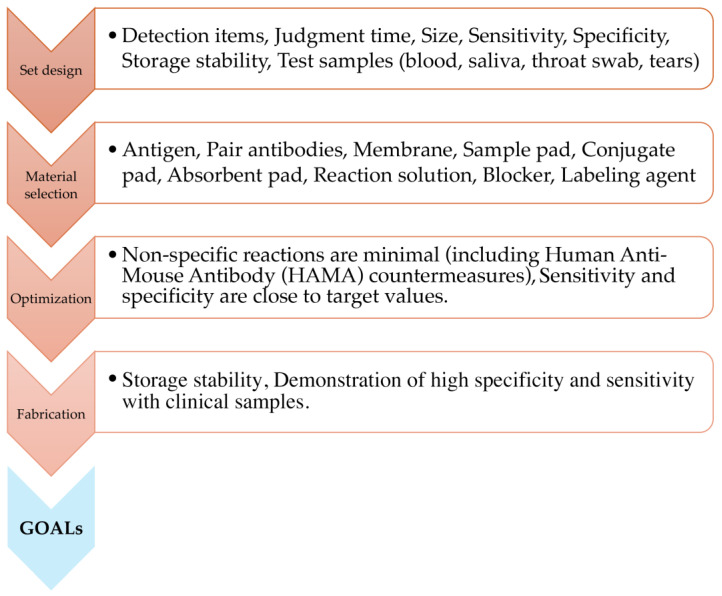
Schematic of lateral flow assay fabrication.

Figure 2 shows the development of the lateral flow assay reagent. The antibodies and antigens vary depending on the target to be measured; therefore, the setting of target values (threshold) and selection of raw materials must be performed carefully. Once the raw materials are determined, it is necessary to confirm that the reaction system of the constructed kit works well, assembles under optimal conditions that minimize non-specific reactions, and demonstrates good stability with high specificity and sensitivity [24]. Immunoassay test papers are typically stored in plastic housings. This prevents exposure of the reagent area and reduces contact opportunities with the actual specimen, thereby reducing the risk of infection exposure to the user and preventing misuse of the kit.

## 3. Pros and Cons of the LFA

The LFA is a highly versatile platform for monitoring nucleic acids, proteins, drugs, toxins, and infectious diseases. Table 1 lists the current features and challenges of typical LFA reagents. Although these are convenient immunoassay reagents, the LFA has unique drawbacks. While conventional LFAs excel in the ease of use, several technical challenges remain.

Detection sensitivity depends on the target but typically ranges in the order of several hundred pg/mL. Several clinical samples having a cycle threshold value (Ct value) above 30 in quantitative reverse transcription polymerase chain reaction (qRT-PCR) and are difficult to detect with the LFA’s current sensitivity. This leads to frequent false negatives because of low antigen concentrations. Additionally, invasive sample collection is often required, as low-invasive samples are frequently excluded from selection. The LFA cannot be used for rule-out testing owing to its lack of high sensitivity, and testing cannot be conducted in the early stages of infection. Furthermore, as results are judged visually, a lack of objectivity occurs, and results may vary depending on the individual making the assessment. If the sensitivity could be improved by 100 times while maintaining the current operability, the LFA could become revolutionary.

In infectious disease testing, the significance lies in detecting antigens, making qualitative assessments the primary focus. However, for items such as drugs, antibodies, hormones, cardiac markers, and C-reactive protein (CRP), where absolute amounts and regular monitoring are crucial, the ability to quantify is essential. The patient’s condition is inferred from the amount of these substances in the sample, making quantification a necessary function. As sample quantification requires normalization with a calibration curve using standard substances, applying this to the current LFA is difficult. Additionally, the narrow measurement range of the LFA imposes limitations on quantification capabilities.

Owing to the insufficient detection sensitivity, a certain amount of antigen in the sample is required. Therefore, with the current LFA, the maximum number of parameters that can be measured on a single test strip is limited to three. If more parameters need to be measured, multiple test strips are required. Visual interpretation also necessitates a certain size, and as the number of items increases, the number of drops and handling required also increases, thereby reducing operability.

Despite these drawbacks, the LFA has significant advantages overcompensating for its shortcomings. Owing to these merits, active development and research are being conducted to address the shortcomings of new LFA technologies (Table 1).

## 4. Efforts towards the Development of New LFA: Development of High-Performance Antibodies

When immunoassay technologies using antibodies were initially reported, polyclonal antibodies were mainly used to detect targets. However, these antibodies, which depend on the immune response of the host or immunized individual, present challenges owing to their unstable production period, quantity, and quality. A famous solution to these problems was reported in 1975 by Köhler and Milstein, who introduced a technique for producing monoclonal antibodies that involved obtaining a single type of antibody molecule [25]. Monoclonal antibodies react with a single antigenic determinant, allowing the production of antibodies with high affinity and specificity. Since the introduction of monoclonal antibodies into immunoassay reagents, their performance has significantly stabilized. Most antibodies used in current LFAs are derived from mice. The monoclonal antibody technology introduced by Köhler is mouse-derived, but antibodies derived from other species, such as camels [26,27], ostriches [28], sharks [29,30], and rabbits [31,32], have also been discovered.

In particular, heavy-chain antibodies, known as nanobodies, are being explored for diagnostics. Heavy-chain antibodies recognize concave epitopes (unique, hard-to-reach parts of the antigens) and are stable because of their smaller size [33,34,35,36,37,38]. In addition, they are easily modified by protein alterations. These characteristics are expected to enable the development of new assay systems that target antigens that are undetectable by traditional antibodies. Additionally, antibodies from higher organisms like rabbits, which have larger and more diverse complementarity determining regions (CDR) than mouse antibodies, are expected to have even greater affinity and specificity.

Although challenges remain, such as the instability of survival after cell fusion in rabbits and humans, myeloma cells that serve as fusion partners have also been developed, and the technology to obtain monoclonal antibodies using the hybridoma method has been established [39,40]. Technologies that directly produce antibodies from B cells without immortalization have also been reported [41,42]. When producing antibodies directly, not from hybridoma cells, it is necessary to produce antibodies using genetic engineering techniques [43]. The advantages of storing and utilizing antibody information in DNA, rather than antibody-producing cells, such as hybridomas, include the following: ① no need to freeze biological samples, ② reduced risk of accidental loss due to disasters or researchers’ decisions, ③ no limit on storage space, and ④ reproducibility and verification by third parties. Additionally, as a secondary effect, methodologies, such as phage display [44,45,46], are being used for mass screening to acquire higher-affinity antibodies than the existing ones. Recently, artificial molecular evolution techniques based on the introduction of mutations using artificial intelligence (AI) have shown significant development [47,48].

In research aimed at increasing the sensitivity of immunodiagnostic reagents, the focus is mainly on enhancing the signal, but more importantly, on reducing noise and improving the signal-to-noise ratio. No uniform method exists for noise reduction. Therefore, it is common to identify the causes of noise and address them individually. One noise reduction strategy is the enzymatic removal of the Fc region of antibodies using enzymes such as papain or pepsin; however, as reagents become more sensitive, the uniformity of the quality of the antibodies themselves becomes a very important factor. To ensure the quality of antibodies, the existing method of acid elution (low pH) with protein G purification products causes the antibodies to aggregate [49]. Techniques that allow gentler conditions for fractionation from purification methods that stress the structure of antibodies have also been developed and are expected to spread [49].

The antibodies used in actual products are traditionally obtained by harvesting monoclonal antibodies from mouse ascites. However, this production method is expected to decline due to the animal welfare perspective. In the future, it is expected that antibodies from organisms with higher specificity and affinity than mice will be used. Additionally, antibodies produced using recombinant technology, which ensures consistent quality, will be increasingly used as raw materials in diagnostics [50]. If high-quality, stable antibodies are incorporated into LFAs, highly sensitive products with no lot-to-lot variability are expected.

### 4.1. Efforts towards New LFA Development—About New Detection Methods

A primary challenge of the LFA is improving the detection sensitivity. Developers aim to embody the ideal POCT they envision by adopting approaches to enhance sensitivity. Common to all immunoassays is the detection of antigen–antibody complexes using labels, such as enzymes. Typically, nanoparticles accumulate, and the presence of an antigen is visually determined. As the number of particles varies depending on the amount of the antigen, it is often difficult to secure a visually sufficient quantity at low antigen levels. Therefore, research studies aimed at enhancing sensitivity has been conducted, and a commonality among them is the amplification of the signal itself. Let us now explore the signal amplification reactions considered to date (Table 2).

### 4.2. Enhancing Sensitivity via Catalytic Detection Principle and Amplification Reactions

**Figure 3:**
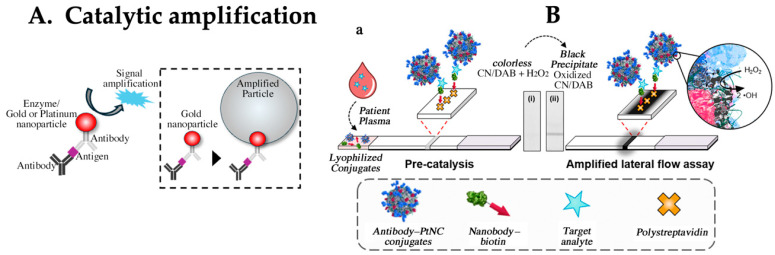
(**A**) Principle of catalytic signal amplification. (**B**) Design concept of catalytic amplification LFA. (**a**) Scheme showing amplified lateral flow immunoassays, where functionalized platinum core–shell nanocatalysts (PtNCs) and biotinylated nanobody fragments are mixed with a plasma or serum sample. In the presence of a target, PtNCs become biotinylated through complexation with the target, and rapid high affinity biotin–streptavidin binding enables a [target-] dependent deposition of PtNCs at the test line. PtNCs bound at the test line catalyze the oxidation of CN/DAB (4-chloro-1-naphthol/3,3′-diaminobenzidine, tetrahydrochloride) substrate in the presence of hydrogen peroxide, producing an insoluble black product that is clearly visible with the naked eye. Figure adapted and modified from ACS Nano 2018, 12, 1, 279–288. Publication date: 7 December 2017. https://doi.org/10.1021/acsnano.7b06229, accessed on 22 July 2024. Copyright © 2017 American Chemical Society. This is an open access article under the CC-BY license. Used under CC-BY. Ref. [63].

The conventional approach involves signal amplification using a catalyst (Figure 3A). One method is based on enzyme amplification, with horseradish peroxidase (HRP) being the primary enzyme, although others, such as alkaline phosphatase (ALP) and cholinesterase, are also used [51,52,53]. The advantage of incorporating enzyme-labeled antibodies into the LFA, similar to ELISA, is that it can also provide quantification [54,55]. This is particularly attractive, as it can deliver results in approximately 20 min compared to the usual period of more than three hours required for quantitatively necessary tests. In addition, sensitivity improvement ranging from a few times to up to 100 times is expected, depending on the particular context and methodology [56,57,58]. However, considering the washing process and substrate solution deployment, more than two steps are required. In addition, considering transport and storage stability, quality assurance of liquid reagents (substrate solutions) could become a bottleneck. Therefore, commonly used labels in the market often involve nanoparticles, such as metal colloids.

Another catalytic approach that has been explored involves the use of gold or platinum particles for signal amplification [59,60]. LFAs using these catalytic effects have been considered to enhance sensitivity and are capable of detecting antigens down to the femtomolar (fM) range (Figure 3B) [61,62,63]. However, particles inevitably remain on the membrane, which can amplify noise-derived signals owing to the catalytic action of the residual particles, making it difficult to differentiate the true reactions. In addition, maintaining the catalytic activity of particles is necessary for practical applications. If a high protein concentration is present on the particle surface, the catalytic sites should not be obscured by the protein. Typically, during blocking treatments, high concentrations of proteins are used to mask labeled particles to prevent nonspecific binding. This treatment can also mask catalytic sites, potentially reducing their activity. Owing to these constraints, several challenges must be overcome before practical implementation.

Noble metal nanoparticles, such as gold nanoparticles (AuNPs), exhibit exceptional optical properties [64] (e.g., exceeding 10^10^ M^−1^ cm^−1^ for 40 nm-AuNPs). Furthermore, depending on the particle size, they exhibit strong optical signal transduction [65]. Based on these characteristics, a two-step amplification approach was adopted to increase the particle size and improve the sensitivity of LFAs [66]. Specifically, gold nanoparticles approximately 10–40 nm in size served as nuclei to which a reducing agent and metal ions were added. The resulting reduced metal particles were deposited onto these nuclei, increasing the size of the labels. Metal ions, including gold [66,67], silver [68], and copper [69] have been investigated as amplification reagents. Silver ion-based amplification methods have been particularly well-studied, achieving sensitivity levels comparable to those of chemiluminescence assays [70,71,72]. A commercial example is the Fujifilm SILVAMP TB lipoarabinomannan (LAM), which utilizes silver amplification to achieve higher sensitivity than conventional LFAs owing to lower antigen levels. This reagent detects LAM, a common glycolipid antigen of Mycobacterium species, in urine [73]. Although enhanced sensitivity was achieved, there are limitations in terms of operability and reaction time. Despite the slightly reduced operability of the two-step amplification reactions, they still achieved higher-sensitivity detection. This potentially allows access to less-invasive samples and broadens the range of measurable targets.

### 4.3. LFA Using Raman Scattering Phenomenon

In addition to their catalytic properties, metal nanoparticles also possess excellent characteristics. Typically, the collective oscillations generated by electromagnetic waves (e.g., light) resonating with electric field vibrations on a metal surface are called surface plasmon resonance (SPR). The disturbance in the equilibrium of free electrons leads to a collective vibrational motion driven by the Coulomb forces acting between them, termed plasma oscillations, and their quantized form is known as plasmons. Propagating surface plasmons on thin-film surfaces are induced by evanescent waves, whereas those localized on metal nanoparticles that do not propagate are called localized plasmons, referred to as localized surface plasmon resonance (LSPR). In such nanostructures, resonance occurs almost independent of the incident angle. For example, gold nanoparticles with a diameter of 80 nm exhibit a peak wavelength of 550 nm [65]. As the particle size increased, so did the scattering.

**Figure 4:**
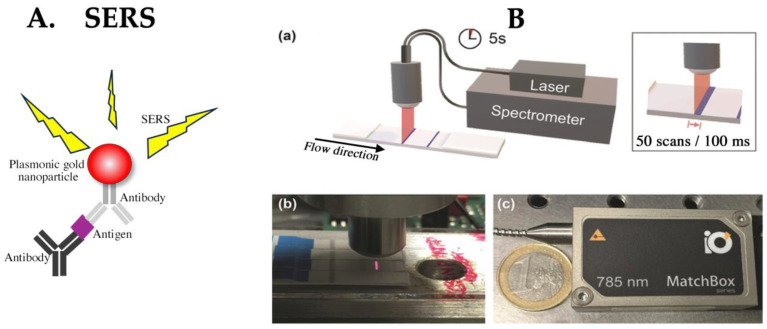
(**A**) The principle of surface-enhanced Raman scattering (SERS). (**B**) A schematic depiction of an LFA based on SERS. (**a**) A schematic representation of a portable Raman reader with an LFA. An averaged Raman spectrum from the entire test line (TL) (approximately 4 mm) is obtained within only 5 s by illuminating along the entire width of the TL with a line focus, and then moving the test strip orthogonally to it using a motorized stage. (**b**) Image of SERS scanning using custom-designed optical fiber probe. (**c**) A photograph of a compact 785 nm diode laser. Figure adapted from Angew. Chem. Int. Ed. 2019, 58, 442–446. Publication date: 5 October 2018. https://doi.org/10.1002/anie.201810917, accessed on 22 July 2024. Copyright © 2018 The Authors. Published by Wiley-VCH Verlag GmbH & Co. KGaA. This is an open access article under the CC BY license. Used under CC-BY. Ref. [81].

In densitometry, metal particles are commonly detected in the test area of the LFA. The use of localized surface plasmons (LSP) is not limited to the scattering and absorption of light but has extensive applications. Local electric field enhancement near metal nanostructures can produce highly efficient Raman scattering, known as surface-enhanced Raman scattering (SERS) (Figure 4A). The initiation of this Raman scattering phenomenon was reported in 1974 by Fleischmann et al., who detected strong Raman scattering from pyridine molecules on silver surfaces [74]. Typically, Raman scattering is extremely weak, requires a long measurement time, and is often masked by background noise. Methods using metal particles for surface-enhanced Raman scattering have been studied since the 1970s. The degree of enhancement varies with the structure and size of the metal nanoparticles. Under optimized conditions, SERS has potential for ultrasensitive detection, even for single molecules [75,76]. To generate Raman-scattered light, it is necessary to create hot spots on the particle surface where the electric field is intense. Particles embedded with the commonly used Raman-active reporter, mercaptobenzoic acid, and used as labels were implemented in the LFA. The anticancer drug 5-fluorouracil has previously been detected in concentrations as low as 4.4 pg/mL [77]. Other applications of this detection method in the LFA include antibody tests and toxin detection (limit of detection (LoD): 0.12 pg/mL) [78,79]. Recently, portable detection devices have been studied, suggesting that such detection systems are suitable for practical use [80,81] (Figure 4B). However, the complexities of particle synthesis and conjugate reagent production may pose challenges for future commercialization.

### 4.4. LFA Based on Heat Detection

**Figure 5:**
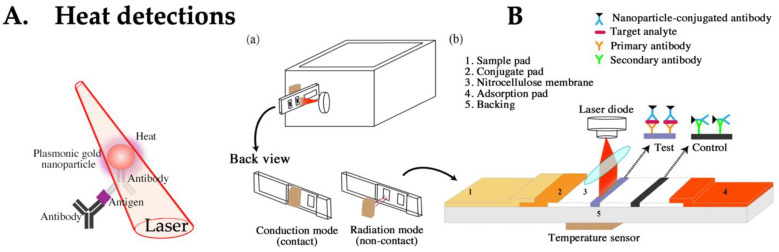
(**A**) The principle of heat detection for LFA. (**B**) Schematic diagram of the plasmonic thermal sensing for the LFA. (**a**) The model of a portable device and the main components (**top**) with two different sensing modes (**bottom**). (**b**) Schematic showing an LFA with thermal sensing setting. Figure adapted from Nanoscale Research Letters 2020,15,10. Publication date: 13 January 2020. https://doi.org/10.1186/s11671-019-3240-3, accessed on 22 July 2024. This is an open access article under the CC-BY license. Used under CC-BY. Ref. [85].

Near-field enhancement by LSPR is used not only in SERS but also in fundamental research related to photonic force (controlling nanoparticles with light) and refractive index sensing [82,83]. Previous studies have shown that performance declines owing to thermal losses; hence, efforts have been made to suppress heat generation (Figure 5A) [84]. However, if the heat source generated from LSPR can be amplified and detected, it can be applied for highly sensitive detection. LFAs that detect the heat emitted from gold particles have achieved sensitivity levels 5–12 times higher than those of visual methods [85,86]. The reaction field uses a membrane composed primarily of combustible nitrocellulose (Figure 5B). Therefore, deliberately generating a heat source may pose commercial limitations, suggesting that the adoption of this detection system may be limited.

### 4.5. LFA Based on Electrochemical Detection

**Figure 6:**
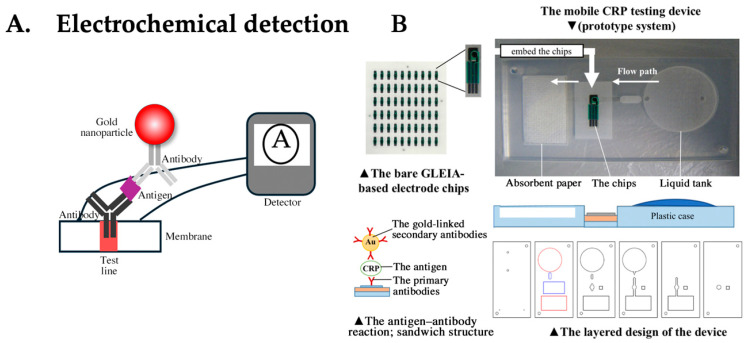
(**A**) The principle of electrochemical (EC) detection. (**B**) Structure of EC-LFA applied for C-reactive protein (CRP) detection, showing the production process from the chips to the prototype and the schematic of gold-linked electrochemical immunosorbent assay (GLEIA) measurement. The mobile CRP-testing device (prototype system) was embedded with the GLEIA-based electrode chips and the liquid tank. The diagram depicting the reaction occurring on the chip is shown at the lower left. The cross-section of the plastic case is shown at the lower right. Adapted from JMIR mHealth uHealth. 2020 Sep 7;8(9): e18782. Publication date: 7 September 2020. https://doi.org/10.2196/18782, accessed on 22 July 2024. Copyright © 2020 JMIR mHealth and uHealth. This is an open access article under the CC-BY license. Used under CC-BY Ref. [88].

Several approaches have incorporated electrochemical detection into the LFA [87]. These methods are known as electrochemical lateral flow assay (EC-LFA)(Figure 6A). The advantages of using electrical signals as detection signals include enhanced sensitivity, expanded dynamic range, quantification capability, ease of miniaturization, and absence of waiting time, as detection is performed in real time. Technical challenges, such as their small size and low cost, are also associated with electrode production; however, electrodes are now fabricated using screen-printing technology and are disposable, overcoming these challenges. EC-LFA, which performs electrical detection, is being commercialized by companies such as iMMUNOSENS. This assay uses gold particles and is called gold-linked electrochemical immunoassay (GLEIA). Since proteins are typically insulating, detecting them in the LFA with protein-coated gold particles is challenging. However, a technique has been established that involves applying voltage to labels, such as gold, causing electron transfer on the electrode to detect targets. The current value is correlated with the amount of antigen, allowing for quantitative measurements. There are reports of achieving target detection with just 1 μL of blood and approximately 1 min for CRP detection [88] (Figure 6B). The EC-LFA, which performs detection using flowing electricity, uses electrodes. If high concentrations of protein are present on the electrode, detection becomes difficult because of protein adhesion to the base. However, innovations in detection technologies using gold particles have overcome these technical barriers. The commercialization and widespread adoption of this technology are highly anticipated.

### 4.6. LFA Using Magnetic Particles

**Figure 7:**
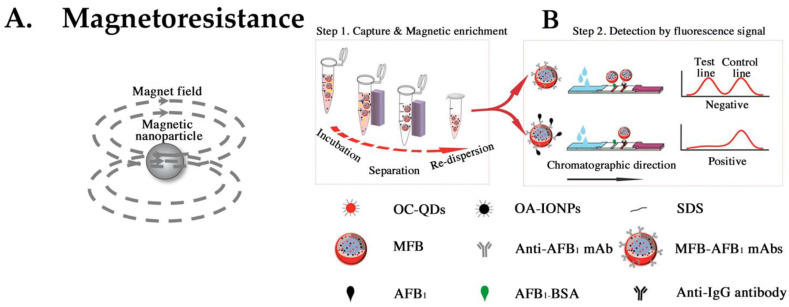
(**A**) Principle of magnetoresistance. (**B**) Principle of the MFB-based immunochromatography assay using the capture, magnetic enrichment, and detection by fluorescence signal. Step 1: Capture and magnetic enrichment. Step 2: Detection by fluorescence signal. Octadecylamine-coated CdSe/ZnS quantum dots (OC-QDs), magnetic fluorescence bead (MFB), aflatoxin B_1_ (AFB_1_), oleic acid-modified iron oxide nanoparticles (OA-IONPs), bovine serum albumin (BSA), sodium dodecyl sulfate (SDS), monoclonal antibodies (mAbs). Figure adapted from Anal. Chem. 2019, 91, 7, 4727–4734 Publication date: 6 March 2019. https://doi.org/10.1021/acs.analchem.9b00223, accessed on 22 July 2024. Copyright © 2019 American Chemical Society. Used with permission from American Chemical Society. Ref. [95].

Superparamagnetic iron oxide nanoparticles, including maghemite and magnetite, are biocompatible and relatively easy to synthesize, which is why they are widely used in the biomedical field as magnetic nanoparticles (MNPs) (Figure 7A). There are various methods for the synthesis of magnetic particles, including thermolysis, reduction, reverse micelle, sonochemical (a method that uses ultrasound to enhance or accelerate chemical reactions), and hydrothermal methods [89]. One significant advantage of MNPs is their ability to combine visual and magnetic detection. Unlike luminescent and fluorescent substrates, they do not degrade easily. Magnetic detection-based LFAs have already been commercialized by several manufacturers, including MagnaBioScience and Magnasense. Preliminary research suggests that the LoD is nearly equivalent to that of visual detection [90,91]. Magnetic-particle detection often uses magnetoresistive sensors. Superconducting quantum interference device magnetometers are used for highly sensitive magnetic measurements [92]. The detection of IgE antibodies at the 2 attomole level was successfully achieved [93]. However, one drawback is that these detectors are expensive.

To leverage the inherent properties of magnetic particles, methods have been reported to enhance the sensitivity by enriching the analyte [94]. This approach is considered essential for achieving high sensitivity in immunoassays.

Recently, bifunctional particles that combine magnetic properties with quantum dots have been developed. This approach involves initial enrichment of the target analytes using MNPs. In the next step, fluorescence is emitted from the quantum dots, and this energy is detected. This system has a sensitivity of several picograms per milliliter (Figure 7B) [95].

### 4.7. LFA Based on Detection of Reflected Electrons

**Figure 8:**
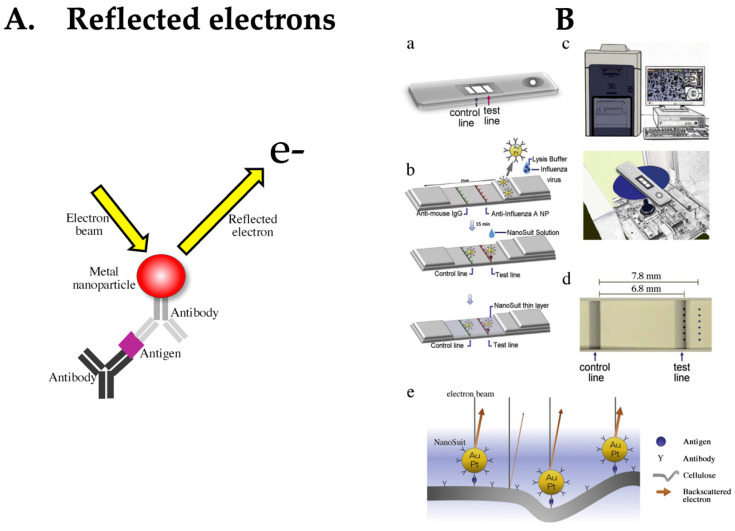
(**A**) Principle of reflected electrons detection. (**B**) Schematic diagram of the NanoSuit methods for LFA using a scanning electron microscope (SEM). (**a**) Structure of rapid test. (**b**) Schematic diagram of the gold/platinum (Au/Pt)-Ab conjugate-linked rapid antigen test. The immune complex reacts with the anti-influenza A nucleoprotein (NP) at the test line (TL) and anti-mouse IgG at the control line (CL). (**c**) Desktop SEM (miniscope TM4000plus; top) used for imaging. Placement of the test strip in the scanning electron microscope chamber (bottom). (**d**) Measurement point. Each of the six points were selected in the TL and background (BG) areas. (**e**) Schematic of backscattering electrons from Au/Pt particles and the cellulose surface coated with the NanoSuit layer. Figure adapted from Journal of Pharmaceutical and Biomedical Analysis, Volume 196, 2021, 113924. Publication Date: 26 January 2021. https://doi.org/10.1016/j.jpba.2021.113924, accessed on 22 July 2024. Copyright © 2021 The Authors. Published by Elsevier B.V. This is an open access article under the CC BY license. Used under CC-BY. Ref. [96].

Finally, we introduce a detection method using reflected electrons. To date, detection methods based on signal amplification that utilize the properties of metal nanoparticles have been reported. The main reason for choosing this amplification method was that the labels were invisible. Recently, the research and development of highly sensitive measurement methods using direct detection systems without amplification reactions has been attempted (Figure 8A). Kawasaki et al. achieved detection sensitivity comparable to that of the qRT-PCR method by combining a conventional LFA with a relatively inexpensive and easy-to-use tabletop scanning electron microscope (SEM), improving detection sensitivity by 73% [96,97] (Figure 8B). The detection system uses a biocompatible liquid called NanoSuit liquid. This special liquid was used to directly detect metal particles on a membrane [98]. The NanoSuit liquid not only imparts conductivity to the LFA device but also protects it, making it easier to reflect electrons from the metal particles. As a result, the background between the membrane support and the label became clear, allowing for sharp imaging of the gold particles. This liquid also exhibits a washing effect. Typically, metal particles at concentrations not visible to the eye can be directly visualized, leading to enhanced sensitivity. As challenges for future commercialization, it is hoped that tabletop SEMs will become portable and that studies of the full automation of measurements will be considered.

In conclusion, we have introduced studies based on the main physical laws of detection systems. There is also ongoing research that attempts to combine detection systems with smartphones and diagnostic apps [99,100]. However, there are many challenges, such as differences in camera performance owing to different smartphone manufacturers, management of OS updates by each manufacturer, color correction (in the case of optical detection), and removal of shadows on the subject during photo shooting. Therefore, in the current situation, either the smartphone itself must be used as a dedicated measuring device, or the measurement accuracy must be ensured using an external accessory.

## 5. Conclusions

The sensitivity of immunochromatographic assays is fundamentally limited by several factors. First, the binding efficiency of antibodies, while crucial for specificity, faces inherent limitations in terms of affinity. Additionally, non-specific binding and background noise present ongoing challenges in minimizing signal interference. The detection systems, including gold nanoparticles and enzyme labels, have physical sensitivity thresholds, and while advanced amplification methods can enhance signals, these also introduce cost and complexity.

Furthermore, the concentration of target antigens within samples can impose a detection limit, especially at extremely low levels. Finally, the chromatographic medium, such as nitrocellulose, restricts both the flow dynamics and the uniformity of reagent migration, thus limiting overall assay sensitivity. Despite recent advancements, such as the application of nanotechnology and digital detection, further improvements in sensitivity must carefully balance reproducibility and cost-effectiveness.

Despite these challenges, what might the future of the LFA and its surrounding environment look like? Although the basic principles of the LFA are unlikely to change significantly, several improvement challenges remain. To enhance the sensitivity of LFAs by leveraging antibody characteristics and combinations from existing research, the following advanced strategies can be considered:

1. Improvement of positive concordance rate using two antibodies. This approach involves incorporating antibodies that specifically react with two or more different antigens expressed by the target into the immunoassay [101]. By utilizing specific antibodies against antigens expressed by different subtypes of a pathogen, this strategy is expected to facilitate the detection of pathogens with multiple subtypes. Additionally, as multiple antigens become the detection targets, the overall target amount for detection is increased (although the antigen amount itself cannot be amplified).
2. Bispecific monoclonal antibodies were proposed as the second generation of monoclonal antibodies, with initial reports from Suresh et al. in the 1980s [102]. These bispecific antibodies independently recognize two different antigens. Given the current advancements in recombinant technology, early application of these antibodies is anticipated. The first advantage is that a single antibody can target twice the number of detection targets. The second advantage lies in using two independent paratopes: while one binds to the antigen, the other binds to a signal amplification substance, such as an enzyme. This method may provide high sensitivity through a secondary reaction with enzyme amplification when particle-based detection alone is insufficient [103].
3. Sensitivity enhancement using secondary antibodies. Typically, the antibody paired in a sandwich assay is labeled with a marker. Instead of this labeled antibody, sensitivity improvements can sometimes be attempted by using highly binding substances, like protein G/A or streptavidin. Additionally, although the reproducibility may be lower, polyclonal antibodies or other multivalent antibodies may improve the sensitivity, as opposed to using monoclonal antibodies.

In addition, both conventional and new LFAs are likely to be integrated into miniaturized devices or portable measuring machines. This is thought to be based on the increasing user needs for higher sensitivity, multiplexing, and objective judgment. However, simply offering higher sensitivity and multiplexing does not lead to explosive popularity. It is important to provide users with the choice (flexibility) to decide upon the best option. One aspect is that it allows users to choose high-sensitivity detection in situations where necessary, which is crucial. Generally, during a viral infection, the virus multiplies rapidly within the body. It is presumed that, among LFA users, a sufficient amount of antigen is often visible through visual judgment. It is often said that LFA only has 70–80% of the sensitivity of PCR. Conversely, the remaining 20–30% minority represents the target group for high-sensitivity detection. As the antigen level is not known in advance, doctors provide the best tests to patients based on their clinical symptoms and experience. If high-sensitivity processing is deemed necessary, with a great reagent, it would be possible to selectively use high-sensitivity detection as needed after testing a strip.

Another perspective involves selective multiplex detection systems. It would be better if users had more freedom to choose the number of measurements and items themselves, rather than just predefined ones. Simultaneous multi-item measurements and situations in which one may want to detect multiple individuals simultaneously for a single item should be considered. Considering infectious diseases as an example, these diseases are seasonal, and their prevalence varies. If there is difficulty in decision-making considering prior probability, having a freely selectable multiplex measurement could maximize its benefits. Finally, as something that will have a value for future society, “information” can be considered. This can be rephrased as giving the best possible choice as far as one can think of one’s own decision-making. It would be ideal if measurement machines, not only dedicated to LFA measurement but also capable of various types of analysis, could manage data, make predictions, and integrate diagnostic results, treatment methods, and coping strategies input into various regional devices. If the diagnostics and effective treatment methods suggested by AI based on these aggregated data could be output, it would be ideal for POCT (e.g., AI Service for Influenza Forecasting., https://www.hitachihyoron.com/rev/archive/2020/r2020_05/05b08/index.html, accessed on 22 July 2024). The LFA is an immunoassay that embodies the concept of POCT. Future high-performance LFAs will allow users, such as nurses and doctors (and operators, such as lab technicians), to obtain complex analysis results at the patient’s bedside within minutes, which would otherwise only be available in the laboratory. The rise of advanced LFAs will not only save time in sending samples to central laboratories but also minimize the TAT for diagnosis and monitoring. Consequently, this will maximize the synergy between proper medical intervention by the doctor and the expected turning point for the patient.

Improvements in the sensitivity of LFAs have led to broader target-detection capabilities, allowing for testing with smaller sample volumes. This, in turn, enables simultaneous multi-target detection. Furthermore, the introduction of automated systems can help mitigate interlot and interfacility variabilities. The future of LFA products is expected to advance towards high-sensitivity and multiplexing capabilities.

Finally, the effectiveness of a test is realized by understanding and using the parameters of the reagent, such as sensitivity and specificity, based on prior probability. In other words, it depends on how it is used.

## Figures and Tables

**Table 1:** Pros and cons of conventional LFA.

Items	Conventional LFA	Disadvantages
Antibody	Monoclonal antibody Polyclonal antibody	Variable quality High sensitivity to pH Binding efficiency varies
Membrane	Nitrocellulose membrane	Single channel pH sensitivity Delicate
Labels	Colloidal gold Fluorescent particles	Stability issues Difficulties in signal amplification Color fading Signal quenching
Readers	Visual judgment sufficient for most applications	Subject to user bias Low precision Need for specialized hardware and software

**Table 2:** Summary of the novel LFA technologies and their disadvantages.

Section	LFA Technology	Disadvantages
4.2	Catalytic Signal Amplification	Operability limitations and longer reaction time
4.3	Raman Scattering Phenomenon	Complex particle synthesis and conjugate reagent production
4.4	Heat Detection	Combustible nitrocellulose limits commercial use
4.5	Electrochemical Detection	Protein adhesion complicates detection
4.6	Magnetic Particles	High cost of detectors
4.7	Reflected Electron Detection	Detectors lack portability and full automation

## Data Availability

Not applicable.
